# 3-[(*E*)-(4-Ethyl­phen­yl)imino­meth­yl]benzene-1,2-diol

**DOI:** 10.1107/S1600536809029924

**Published:** 2009-07-31

**Authors:** Zeynep Keleşoğlu, Orhan Büyükgüngör, Çiğdem Albayrak, Mustafa Odabaşoğlu

**Affiliations:** a Department of Physics, Ondokuz Mayıs University, TR-55139 Samsun, Turkey; b Sinop University, Sinop Faculty of Education, Sinop, Turkey; c Pamukkale University, Denizli Technical Vocational School, Denizli, Turkey

## Abstract

The title compound, C_15_H_15_NO_2_, adopts the enol–imine tautomeric form. The dihedral angle between the two benzene rings is 48.1 (1)°. Intra­molecular O—H⋯N and O—H⋯O hydrogen bonds generate *S*(6) and *S*(5) ring motifs, respectively. In the crystal, mol­ecules are linked into centrosymmetric *R*_2_^2^(10) dimers *via* pairs of O—H⋯O hydrogen bonds and the dimers may interact through very weak by π–π inter­actions [centroid–centroid distance = 4.150 (1) Å]. The ethyl group is disordered over two orientations, with occupancies of 0.587 (11) and 0.413 (11).

## Related literature

For the photochromic and thermochromic properties of Schiff base compounds, see: Elmali *et al.* (1999 ▶); Guha *et al.* (2000 ▶); Kletski *et al.* (1997 ▶); Kownacki *et al.* (1994 ▶); Zgierski *et al.* (2000 ▶). For Schiff base tautomerism, see: Alarcon *et al.* (1995 ▶); Dudek *et al.*, (1966 ▶); Salman *et al.* (1991 ▶, 1993 ▶). For a related structure, see: Özek *et al.* (2009 ▶). For graph-set motifs, see: Bernstein *et al.* (1995 ▶).
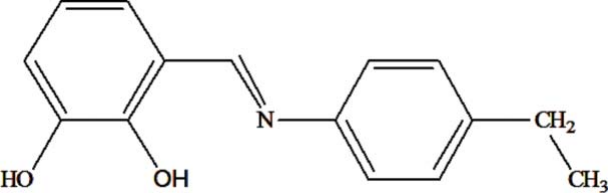


## Experimental

### 

#### Crystal data

C_15_H_15_NO_2_
*M* *_r_* = 241.28
Triclinic, 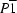

*a* = 6.1893 (4) Å
*b* = 8.7704 (6) Å
*c* = 12.7605 (9) Å
α = 87.326 (6)°
β = 86.397 (6)°
γ = 69.394 (5)°
*V* = 646.85 (8) Å^3^
*Z* = 2
Mo *K*α radiation
μ = 0.08 mm^−1^
*T* = 296 K
0.54 × 0.41 × 0.31 mm

#### Data collection

Stoe IPDS II diffractometer
Absorption correction: integration (*X-RED32*; Stoe & Cie, 2002 ▶) *T*_min_ = 0.966, *T*_max_ = 0.979
8683 measured reflections
2668 independent reflections
1896 reflections with *I* > 2σ(*I*)
*R*_int_ = 0.042

#### Refinement

*R*[*F* ^2^ > 2σ(*F* ^2^)] = 0.051
*wR*(*F* ^2^) = 0.148
*S* = 1.03
2668 reflections
191 parameters
28 restraints
H atoms treated by a mixture of independent and constrained refinement
Δρ_max_ = 0.24 e Å^−3^
Δρ_min_ = −0.13 e Å^−3^

### 

Data collection: *X-AREA* (Stoe & Cie, 2002 ▶); cell refinement: *X-AREA*; data reduction: *X-RED32* (Stoe & Cie, 2002 ▶); program(s) used to solve structure: *SHELXS97* (Sheldrick, 2008 ▶); program(s) used to refine structure: *SHELXL97* (Sheldrick, 2008 ▶); molecular graphics: *ORTEP-3 for Windows* (Farrugia, 1997 ▶); software used to prepare material for publication: *WinGX* (Farrugia, 1999 ▶).

## Supplementary Material

Crystal structure: contains datablocks I, global. DOI: 10.1107/S1600536809029924/ci2862sup1.cif

Structure factors: contains datablocks I. DOI: 10.1107/S1600536809029924/ci2862Isup2.hkl

Additional supplementary materials:  crystallographic information; 3D view; checkCIF report

## Figures and Tables

**Table 1:** Hydrogen-bond geometry (Å, °)

*D*—H⋯*A*	*D*—H	H⋯*A*	*D*⋯*A*	*D*—H⋯*A*
O1—H1⋯N1	0.95 (3)	1.72 (3)	2.596 (2)	152 (2)
O2—H2⋯O1	0.88 (3)	2.29 (3)	2.7307 (19)	111 (2)
O2—H2⋯O1^i^	0.88 (3)	2.06 (3)	2.818 (2)	143 (2)

Symmetry code: (i) 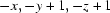.

**Fig. 1.**
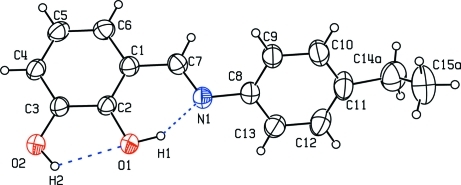
The molecular structure of (I), showing 30% probability displacement ellipsoids and the atom-numbering scheme. Only the major disorder component of the ethyl group is shown. Dashed lines indicate hydrogen bonds.

**Fig. 2.**
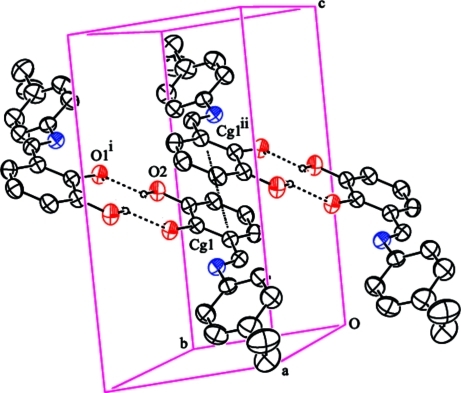
A packing diagram for (I), showing the formation dimers through O—H···O hydrogen bonds and π–π interactions. [Symmetry code: (i) -x, 1 - y, 1 - z; (ii) 1 - x, 1 - y, 1 - z]. H atoms not involved in hydrogen bonding (dashed lines) have been omitted for clarity. Cg1 and Cg2 are centroids of the C1-C6 and C8-C13 rings, respectively.

## supplementary crystallographic information

### Comment

There has been considerable interest in some Schiff bases derived from salicylaldehyde and substituted salicylaldehyde because they show thermochromism and photochromism in the solid state (Kownacki *et al.*, 1994). The tautomerism in the Schiff base ligands plays an important role for distinguishing their photochromic (Guha *et al.*, 2000) and thermochromic (Zgierski *et al.*, 2000) characteristics. It has been proposed that molecules showing thermochromism are planar, while those showing photochromism are non-planar (Kletski *et al.*, 1997), both phenomena being associated with a proton transfer (Elmali *et al.*, 1999). Schiff bases derived from the condensation of salicylaldehyde with aniline and substituted aniline, and naphthaldehyde with aniline exists as enol-imine (Dudek *et al.*, 1966), keto-amine (Salman *et al.*, 1991), or enol-imine/keto-amine form (Salman *et al.*, 1993; Alarcon *et al.*, 1995) in all solvents.

The X-ray analysis shows that the title compound prefers an enol-imine tautomeric form, with a strong intramolecular O1—H1···N1 hydrogen bond. This is also confirmed by the C2—O1 [1.361 (2) Å], C7—N1 [1.278 (2) Å], C1—C7 [1.445 (2) Å] and C1—C2 [1.399 (2) Å] bond lengths (Fig. 1). The C2—O1 bond length of 1.361 (2) Å indicates a single-bond character and the C7—N1 bond length of 1.278 (2) Å indicates a high degree of double-bond character. Similar results were observed for (*E*)-4-methoxy-2-[(*o*-tolilimino)methyl]phenol [C—O = 1.357 (2) Å, C═N= 1.286 (2) Å; Özek *et al.*, 2009]. An intramolecular O2—H2···O1 hydrogen bond is also observed. The O—H···N and O—H···O hydrogen bonds generate S(6) and S(5) ring motifs, respectively (Bernstein *et al.*, 1995).

The dihedral angle between benzene rings A(C1-C6) and B(C8-C13) is 48.1 (1)°. The nearly planar S(6) ring C(O1/H1/N1/C1/C2/C7) is oriented with respect to rings A and B at dihedral angles of A/C = 1.89 (42)° and B/C = 46.23 (25)°. It is known that Schiff bases may exhibit thermochromism or photochromism, depending on the planarity or non-planarity of the molecule, respectively. Since the title moleclule is non-planar, one can expect photochromic properties in title compound.

In the crystal structure, molecules are linked into centrosymmetric R_2_^2^(10) dimers via O—H···O hydrogen bonds (Table 2). A very weak π–π interaction occurs between A(C1-C6) rings at (x, y, z) and (1-x, 1-y, 1-z), with a ring centroid-to centroid distance of 4.150 (1) Å; only atoms C1, C2 and C3 are involved in the interactions as the rings are displaced.

### Experimental

Compound (I) was prepared by refluxing a mixture of 2,3-dihidroxy benzalaldehyde (0.5 g 0.0036 mol) in  ethanol (20 ml) and 4-ethylaniline (0.436 g 0.0036 mol) in ethanol (20 ml). The reaction mixture was stirred for 1 h under reflux. Single crystals of (I) suitable for X-ray analysis were obtained by slow evaporation of a methanol solution (yield 87%, m.p. 378-379 K).

### Refinement

The ethyl group is disordered over two orientations, with occupancies of 0.587 (11) and 0.413 (11). The U^ij^ parameters of the disordered atoms were restrained to an approximate isotropic behaviour. The C—C distances involving disordered atoms were restrained to 1.54 (2) Å. The hydroxyl H atoms were located in a difference Fourier map and were refined freely. All other H-atoms were refined using a riding model with d(C-H) = 0.93–0.96 Å (*U*_iso_ = 1.2*U*_eq_ of the parent atom) for aromatic and ethyl C atoms and d(C-H) = 0.97 Å (*U*_iso_=1.5*U*_eq_ of the parent atom) for methyl C atoms.

### Crystal data

**Table:**

C_15_H_15_NO_2_	*Z* = 2
*M**_r_* = 241.28	*F*(000) = 256
Triclinic, *P*1	*D*_x_ = 1.239 Mg m^−^^3^
Hall symbol: -P 1	Mo *K*α radiation, λ = 0.71073 Å
*a* = 6.1893 (4) Å	Cell parameters from 8683 reflections
*b* = 8.7704 (6) Å	θ = 1.6–28.0°
*c* = 12.7605 (9) Å	µ = 0.08 mm^−^^1^
α = 87.326 (6)°	*T* = 296 K
β = 86.397 (6)°	Prism, red
γ = 69.394 (5)°	0.54 × 0.41 × 0.31 mm
*V* = 646.85 (8) Å^3^	

### Data collection

**Table:**

Stoe IPDS II diffractometer	2668 independent reflections
Radiation source: fine-focus sealed tube	1896 reflections with *I* > 2σ(*I*)
graphite	*R*_int_ = 0.042
Detector resolution: 6.67 pixels mm^-1^	θ_max_ = 26.5°, θ_min_ = 1.6°
rotation method scans	*h* = −7→7
Absorption correction: integration (*X-RED32*; Stoe & Cie, 2002)	*k* = −11→11
*T*_min_ = 0.966, *T*_max_ = 0.979	*l* = −15→15
8683 measured reflections	

### Refinement

**Table:**

Refinement on *F*^2^	Secondary atom site location: difference Fourier map
Least-squares matrix: full	Hydrogen site location: inferred from neighbouring sites
*R*[*F*^2^ > 2σ(*F*^2^)] = 0.051	H atoms treated by a mixture of independent and constrained refinement
*wR*(*F*^2^) = 0.148	*w* = 1/[σ^2^(*F*_o_^2^) + (0.0674*P*)^2^ + 0.1042*P*] where *P* = (*F*_o_^2^ + 2*F*_c_^2^)/3
*S* = 1.03	(Δ/σ)_max_ = 0.001
2668 reflections	Δρ_max_ = 0.24 e Å^−^^3^
191 parameters	Δρ_min_ = −0.13 e Å^−^^3^
28 restraints	Extinction correction: *SHELXL97* (Sheldrick, 2008), Fc^*^=kFc[1+0.001xFc^2^λ^3^/sin(2θ)]^-1/4^
Primary atom site location: structure-invariant direct methods	Extinction coefficient: 0.030 (7)

### Special details

**Table:**

Geometry. All e.s.d.'s (except the e.s.d. in the dihedral angle between two l.s. planes) are estimated using the full covariance matrix. The cell e.s.d.'s are taken into account individually in the estimation of e.s.d.'s in distances, angles and torsion angles; correlations between e.s.d.'s in cell parameters are only used when they are defined by crystal symmetry. An approximate (isotropic) treatment of cell e.s.d.'s is used for estimating e.s.d.'s involving l.s. planes.
Refinement. Refinement of *F*^2^ against ALL reflections. The weighted *R*-factor *wR* and goodness of fit *S* are based on *F*^2^, conventional *R*-factors *R* are based on *F*, with *F* set to zero for negative *F*^2^. The threshold expression of *F*^2^ > σ(*F*^2^) is used only for calculating *R*-factors(gt) *etc*. and is not relevant to the choice of reflections for refinement. *R*-factors based on *F*^2^ are statistically about twice as large as those based on *F*, and *R*- factors based on ALL data will be even larger.

### Fractional atomic coordinates and isotropic or equivalent isotropic displacement parameters (Å^2^)

**Table:**

	*x*	*y*	*z*	*U*_iso_*/*U*_eq_	Occ. (<1)
C1	0.5280 (3)	0.5619 (2)	0.34375 (15)	0.0590 (5)	
C2	0.3189 (3)	0.5824 (2)	0.40019 (14)	0.0557 (4)	
C3	0.2107 (3)	0.7228 (2)	0.45769 (15)	0.0583 (5)	
C4	0.3058 (3)	0.8430 (2)	0.45564 (17)	0.0664 (5)	
H4	0.2322	0.9372	0.4932	0.080*	
C5	0.5101 (4)	0.8250 (2)	0.39809 (18)	0.0717 (6)	
H5	0.5719	0.9076	0.3964	0.086*	
C6	0.6212 (4)	0.6859 (2)	0.34379 (17)	0.0700 (5)	
H6	0.7600	0.6736	0.3065	0.084*	
C7	0.6472 (3)	0.4135 (2)	0.28805 (15)	0.0636 (5)	
H7	0.7913	0.3996	0.2554	0.076*	
C8	0.6932 (3)	0.1541 (2)	0.23042 (15)	0.0629 (5)	
C9	0.9261 (4)	0.0745 (3)	0.24457 (18)	0.0752 (6)	
H9	1.0026	0.1169	0.2892	0.090*	
C10	1.0450 (4)	−0.0671 (3)	0.1929 (2)	0.0914 (8)	
H10	1.2010	−0.1202	0.2041	0.110*	
C11	0.9377 (5)	−0.1323 (3)	0.1246 (2)	0.0994 (8)	
C12	0.7038 (5)	−0.0553 (3)	0.1148 (2)	0.0949 (8)	
H12	0.6263	−0.0994	0.0718	0.114*	
C13	0.5816 (4)	0.0857 (3)	0.16706 (18)	0.0789 (6)	
H13	0.4233	0.1347	0.1595	0.095*	
C14A	1.0861 (17)	−0.2683 (8)	0.0432 (7)	0.120 (3)	0.587 (11)
H14A	0.9952	−0.2714	−0.0153	0.144*	0.587 (11)
H14B	1.2235	−0.2474	0.0164	0.144*	0.587 (11)
C15A	1.1473 (16)	−0.4208 (9)	0.1053 (5)	0.143 (3)	0.587 (11)
H15A	1.2155	−0.5112	0.0597	0.214*	0.587 (11)
H15B	1.0107	−0.4296	0.1409	0.214*	0.587 (11)
H15C	1.2558	−0.4216	0.1561	0.214*	0.587 (11)
C14B	1.0391 (18)	−0.3057 (11)	0.0870 (10)	0.116 (4)	0.413 (11)
H14C	1.0239	−0.3787	0.1441	0.139*	0.413 (11)
H14D	0.9493	−0.3172	0.0303	0.139*	0.413 (11)
C15B	1.273 (2)	−0.3543 (17)	0.0517 (11)	0.164 (5)	0.413 (11)
H15D	1.2997	−0.4273	−0.0053	0.246*	0.413 (11)
H15E	1.3686	−0.4085	0.1082	0.246*	0.413 (11)
H15F	1.3090	−0.2601	0.0282	0.246*	0.413 (11)
N1	0.5630 (3)	0.30067 (18)	0.28177 (13)	0.0637 (4)	
O1	0.2152 (2)	0.46864 (15)	0.40254 (11)	0.0659 (4)	
O2	0.0118 (2)	0.74271 (17)	0.51685 (13)	0.0749 (5)	
H1	0.314 (5)	0.386 (3)	0.358 (2)	0.099 (8)*	
H2	−0.027 (5)	0.655 (4)	0.515 (2)	0.111 (9)*	

### Atomic displacement parameters (Å^2^)

**Table:**

	*U*^11^	*U*^22^	*U*^33^	*U*^12^	*U*^13^	*U*^23^
C1	0.0606 (11)	0.0534 (10)	0.0622 (11)	−0.0192 (8)	−0.0047 (9)	0.0012 (8)
C2	0.0577 (10)	0.0463 (9)	0.0641 (11)	−0.0189 (8)	−0.0078 (8)	−0.0009 (7)
C3	0.0546 (10)	0.0491 (9)	0.0695 (12)	−0.0150 (8)	−0.0065 (8)	−0.0049 (8)
C4	0.0681 (12)	0.0502 (10)	0.0818 (13)	−0.0197 (9)	−0.0128 (10)	−0.0079 (9)
C5	0.0766 (13)	0.0580 (11)	0.0896 (15)	−0.0339 (10)	−0.0112 (11)	0.0003 (10)
C6	0.0674 (12)	0.0667 (12)	0.0807 (14)	−0.0301 (10)	−0.0005 (10)	0.0004 (10)
C7	0.0628 (11)	0.0606 (11)	0.0638 (12)	−0.0185 (9)	0.0036 (9)	−0.0008 (9)
C8	0.0712 (12)	0.0564 (10)	0.0585 (11)	−0.0195 (9)	0.0014 (9)	−0.0034 (8)
C9	0.0736 (13)	0.0669 (12)	0.0793 (14)	−0.0156 (10)	−0.0063 (10)	−0.0143 (10)
C10	0.0826 (16)	0.0733 (14)	0.1042 (19)	−0.0078 (12)	−0.0029 (13)	−0.0206 (13)
C11	0.112 (2)	0.0714 (14)	0.104 (2)	−0.0159 (14)	−0.0015 (15)	−0.0307 (13)
C12	0.117 (2)	0.0750 (15)	0.0955 (18)	−0.0321 (15)	−0.0172 (15)	−0.0207 (13)
C13	0.0826 (15)	0.0695 (13)	0.0861 (15)	−0.0265 (11)	−0.0127 (12)	−0.0062 (11)
C14A	0.144 (6)	0.085 (4)	0.129 (5)	−0.042 (4)	0.014 (4)	0.013 (3)
C15A	0.182 (7)	0.098 (5)	0.114 (4)	−0.007 (4)	0.007 (4)	−0.021 (3)
C14B	0.137 (6)	0.059 (5)	0.136 (7)	−0.016 (4)	0.034 (5)	−0.044 (5)
C15B	0.156 (8)	0.135 (7)	0.185 (9)	−0.036 (6)	0.038 (7)	−0.029 (6)
N1	0.0668 (10)	0.0559 (9)	0.0655 (10)	−0.0181 (7)	0.0016 (7)	−0.0054 (7)
O1	0.0642 (8)	0.0517 (7)	0.0840 (10)	−0.0234 (6)	0.0090 (7)	−0.0149 (6)
O2	0.0646 (9)	0.0587 (8)	0.1033 (12)	−0.0236 (7)	0.0119 (7)	−0.0265 (7)

### Geometric parameters (Å, °)

**Table:**

C1—C2	1.399 (3)	C11—C12	1.376 (4)
C1—C6	1.399 (3)	C11—C14B	1.514 (7)
C1—C7	1.445 (3)	C11—C14A	1.603 (8)
C2—O1	1.361 (2)	C12—C13	1.379 (3)
C2—C3	1.395 (2)	C12—H12	0.93
C3—O2	1.364 (2)	C13—H13	0.93
C3—C4	1.375 (3)	C14A—C15A	1.464 (11)
C4—C5	1.385 (3)	C14A—H14A	0.97
C4—H4	0.93	C14A—H14B	0.97
C5—C6	1.367 (3)	C15A—H15A	0.96
C5—H5	0.93	C15A—H15B	0.96
C6—H6	0.93	C15A—H15C	0.96
C7—N1	1.278 (2)	C14B—C15B	1.405 (15)
C7—H7	0.93	C14B—H14C	0.97
C8—C13	1.378 (3)	C14B—H14D	0.97
C8—C9	1.382 (3)	C15B—H15D	0.96
C8—N1	1.419 (2)	C15B—H15E	0.96
C9—C10	1.375 (3)	C15B—H15F	0.96
C9—H9	0.93	O1—H1	0.95 (3)
C10—C11	1.383 (4)	O2—H2	0.88 (3)
C10—H10	0.93		
			
C2—C1—C6	118.93 (17)	C10—C11—C14A	121.0 (4)
C2—C1—C7	120.31 (17)	C11—C12—C13	121.7 (2)
C6—C1—C7	120.76 (18)	C11—C12—H12	119.2
O1—C2—C3	117.70 (17)	C13—C12—H12	119.2
O1—C2—C1	122.45 (16)	C8—C13—C12	120.2 (2)
C3—C2—C1	119.85 (16)	C8—C13—H13	119.9
O2—C3—C4	119.15 (17)	C12—C13—H13	119.9
O2—C3—C2	121.02 (16)	C15A—C14A—C11	104.1 (6)
C4—C3—C2	119.83 (18)	C15A—C14A—H14A	110.9
C3—C4—C5	120.55 (18)	C11—C14A—H14A	110.9
C3—C4—H4	119.7	C15A—C14A—H14B	110.9
C5—C4—H4	119.7	C11—C14A—H14B	110.9
C6—C5—C4	120.14 (19)	H14A—C14A—H14B	109.0
C6—C5—H5	119.9	C14A—C15A—H15A	109.5
C4—C5—H5	119.9	C14A—C15A—H15B	109.5
C5—C6—C1	120.7 (2)	H15A—C15A—H15B	109.5
C5—C6—H6	119.7	C14A—C15A—H15C	109.5
C1—C6—H6	119.7	H15A—C15A—H15C	109.5
N1—C7—C1	122.76 (18)	H15B—C15A—H15C	109.5
N1—C7—H7	118.6	C15B—C14B—C11	114.5 (9)
C1—C7—H7	118.6	C15B—C14B—H14C	108.6
C13—C8—C9	118.79 (18)	C11—C14B—H14C	108.6
C13—C8—N1	118.77 (19)	C15B—C14B—H14D	108.6
C9—C8—N1	122.40 (18)	C11—C14B—H14D	108.6
C10—C9—C8	120.3 (2)	H14C—C14B—H14D	107.6
C10—C9—H9	119.9	C14B—C15B—H15D	109.5
C8—C9—H9	119.9	C14B—C15B—H15E	109.5
C9—C10—C11	121.5 (2)	H15D—C15B—H15E	109.5
C9—C10—H10	119.3	C14B—C15B—H15F	109.5
C11—C10—H10	119.3	H15D—C15B—H15F	109.5
C12—C11—C10	117.4 (2)	H15E—C15B—H15F	109.5
C12—C11—C14B	116.2 (5)	C7—N1—C8	120.19 (17)
C10—C11—C14B	123.6 (5)	C2—O1—H1	103.8 (15)
C12—C11—C14A	120.4 (4)	C3—O2—H2	111.0 (18)
			
C6—C1—C2—O1	178.84 (17)	C9—C10—C11—C12	−3.7 (4)
C7—C1—C2—O1	−2.3 (3)	C9—C10—C11—C14B	−164.1 (6)
C6—C1—C2—C3	−1.9 (3)	C9—C10—C11—C14A	164.1 (4)
C7—C1—C2—C3	177.02 (17)	C10—C11—C12—C13	2.8 (4)
O1—C2—C3—O2	2.1 (3)	C14B—C11—C12—C13	164.7 (6)
C1—C2—C3—O2	−177.26 (17)	C14A—C11—C12—C13	−165.0 (4)
O1—C2—C3—C4	−178.37 (17)	C9—C8—C13—C12	−3.3 (3)
C1—C2—C3—C4	2.3 (3)	N1—C8—C13—C12	179.0 (2)
O2—C3—C4—C5	178.65 (18)	C11—C12—C13—C8	0.6 (4)
C2—C3—C4—C5	−0.9 (3)	C12—C11—C14A—C15A	−109.9 (7)
C3—C4—C5—C6	−0.9 (3)	C10—C11—C14A—C15A	82.7 (8)
C4—C5—C6—C1	1.4 (3)	C14B—C11—C14A—C15A	−21.5 (12)
C2—C1—C6—C5	0.0 (3)	C12—C11—C14B—C15B	151.7 (11)
C7—C1—C6—C5	−178.83 (18)	C10—C11—C14B—C15B	−47.7 (16)
C2—C1—C7—N1	4.6 (3)	C14A—C11—C14B—C15B	45.5 (13)
C6—C1—C7—N1	−176.53 (19)	C1—C7—N1—C8	−176.85 (17)
C13—C8—C9—C10	2.5 (3)	C13—C8—N1—C7	−139.4 (2)
N1—C8—C9—C10	−179.9 (2)	C9—C8—N1—C7	43.0 (3)
C8—C9—C10—C11	1.1 (4)		

### Hydrogen-bond geometry (Å, °)

**Table:**

*D*—H···*A*	*D*—H	H···*A*	*D*···*A*	*D*—H···*A*
O1—H1···N1	0.95 (3)	1.72 (3)	2.596 (2)	152 (2)
O2—H2···O1	0.88 (3)	2.29 (3)	2.7307 (19)	111 (2)
O2—H2···O1^i^	0.88 (3)	2.06 (3)	2.818 (2)	143 (2)

Symmetry codes: (i) −*x*, −*y*+1, −*z*+1.
